# Estimating the Prevalence of Pseudomyxoma Peritonei in Europe Using a Novel Statistical Method

**DOI:** 10.1245/s10434-020-08655-8

**Published:** 2020-06-02

**Authors:** Thale Dawn J. H. Patrick-Brown, Norman John Carr, David M. Swanson, Stein Larsen, Faheez Mohamed, Kjersti Flatmark

**Affiliations:** 1 Department of Tumor Biology, The Norwegian Radium Hospital, Oslo University Hospital Oslo, Oslo, Norway; 2 Department of Oncology, Akershus University Hospital, Lørenskog, Norway; 3 Peritoneal Malignancy Institute, Basingstoke and North Hampshire Hospital, Aldermaston Road, Basingstoke, UK; 4 Oslo Centre for Biostatistics and Epidemiology (OCBE), University of Oslo, Oslo, Norway; 5 Department of Tumor Biology, Institute for Cancer Research, The Norwegian Radium Hospital, Oslo University Hospital, Oslo, Norway; 6 Faculty of Medicine, University of Oslo, Oslo, Norway

## Abstract

**Background:**

The determination of the incidence and prevalence of rare diseases is important for economists and health-care providers. Pseudomyxoma peritonei (PMP) is a rare, slow-growing abdominal cancer that represents a substantial burden on both patients and health-care systems. The incidence rate was previously approximated at 1–2 people per million per year; this incidence has never been challenged, and the prevalence has not been estimated.

**Methods:**

Epidemiological data from Norway and England were obtained and analysed to calculate a minimum incidence rate based on the number of patients having a first surgical intervention for PMP. A novel method was then used to determine a prevalence rate for PMP, incorporating incidence, death, and cure rates in a multi-year analysis that accounted for the increasing population of Europe over a 10-year period.

**Results:**

An incidence rate of 3.2 people per million per year was calculated, with a corresponding estimated prevalence rate of 22 people per million per year. By this calculation, 11,736 people in Europe were estimated to be living with PMP in 2018.

**Conclusion:**

Incidence and prevalence are essential tools for assessment of the financial and human cost of a disease. For rare diseases, such as PMP, the lack of accurate registries presents a particular challenge in determining such health-related statistical parameters. Based on our calculations, a significant number of people are living with PMP in Europe, underlining the need for appropriate resource allocation to ensure that adequate health-care measures are provided.

The estimation of the number of people suffering from rare diseases is important to the health-care community. Statistical indicators help shed light on not only the number of new cases that may present each year (incidence), but also on the number of people who might be affected by the disease at any given time (prevalence). These figures assist clinicians, medical economists and politicians with long-term planning for the use of resources and infrastructure, such as bed and facility planning, staffing, and budgeting. Monitoring of incidence and prevalence is also useful for prevention and treatment strategies, as well as understanding the continuing human cost of the disease. Underestimating incidence and prevalence leads to inadequate provision of support to patients who may require repeated surgical interventions and ongoing medical treatment. This further impacts the economy through people’s inability to work or take an active role in the community due to symptoms and disability.

The monitoring of health-related statistics through national databases lends itself to the accurate calculation of incidence and prevalence, allowing good quality data to be produced for forecasting and planning activities. Due to lack of resources, the demographics of rare diseases can be challenging to determine using standard medical reporting tools, making useful predictive models difficult to establish. Less common diseases may further suffer from lower rates of reporting, under-diagnosis or misdiagnosis, leading to underestimation of both incidence and prevalence.

Pseudomyxoma peritonei (PMP) often called “jelly–belly”, is a rare cancer that generally presents as multifocal mucinous tumours in the abdominal cavity causing increased abdominal girth, pain and pressure on internal organs due to the accumulation of large amounts of mucinous tumour. PMP most commonly originates from tumours of the appendix that rupture, seeding cancerous cells throughout the abdominal cavity via paths of fluid re-absorption. Treatment involves cytoreductive surgery (CRS) and hyperthermic intraperitoneal chemotherapy (HIPEC) which, when performed at specialist centres, can lead to an overall 10-year survival of around 63%.^1^ For patients that cannot be cured by surgery, no effective treatments exist, and since PMP is a slow-growing cancer, patients may live for many years with active disease and worsening symptoms, presenting a substantial burden on the health-care system. Estimation of prevalence is therefore essential for resource allocation.

Prevalence estimates depend on the incidence rate, which for PMP have been widely quoted as approximately 1–2 people per million.^2^ This estimation was made in a study looking at neoplasms of known appendiceal origin and included only the low-grade subtype of PMP, so some cases may have been left out of the calculation. It is widely believed by experts in the field that the total number of incident cases is higher because PMP is often misdiagnosed, leading to an under-reporting of the condition. As formal registries for PMP do not exist, challenges in extrapolating figures have meant that incidence and prevalence have been difficult to calculate.

This paper seeks to determine a scientifically grounded incidence rate based on the number of cases having surgical intervention in one country (Norway) over a 10-year period, validated by data from England. The paper also outlines a novel waterfall method for calculating prevalence in an increasing population and then presents an estimated prevalence of PMP for the European population.

## Materials and Methods

### Patients

To determine the incidence rate of PMP in Norway, data from records of patients treated for PMP between 2009 and 2018 at the Norwegian Radium Hospital, part of Oslo University Hospital Comprehensive Cancer Centre, were obtained. The Norwegian Radium Hospital has performed CRS and HIPEC since 1994, and serves the entire Norwegian population of more than five million through the “Norwegian National Unit for Hyperthermic Intraperitoneal Chemotherapy in Colorectal Cancer, Pseudomyxoma Peritonei and Abdominal Mesothelioma”. Data for England included patients treated for PMP at the Peritoneal Malignancy Institute, Basingstoke, between 2012 and 2018, who lived in the UK regions of London, the South East, and South West of England, East and West Midlands, Wales, Guernsey, Jersey and the East of England. All PMP cases were verified by an expert pathologist.

### Population Data

European population statistics for the years 1989–2018 were obtained from the United Nations’ Department of Economic and Social Affairs Population Division,^3^ which provides population estimates using various time-tested methods, described in their manual.^4^ Overall population numbers were identified for Austria, Belgium, Bulgaria, Croatia, Cyprus, Czech Republic, Denmark, Estonia, Finland, France, Germany, Greece, Hungary, Ireland, Italy, Latvia, Lithuania, Luxembourg, Malta, the Netherlands, Norway, Poland, Portugal, Romania, Slovakia, Slovenia, Spain, Sweden and the United Kingdom (hereafter referred to as Europe). Annual population estimates for administrative regions within England were obtained from reports produced by the UK Office of National Statistics.^5^

### Surveillance Period

To determine the appropriate length of time to consider a patient under active surveillance (with or without active disease), articles reporting survival parameters after surgery for PMP were consulted. Published PMP survival curves appear to plateau by year 10, and almost all recurrences (98.7%) are reported to have occurred by that time.^6^–^16^ A surveillance period of 10 years was therefore adopted to calculate the period prevalence.

### Annual Death Rates

Due to the length of the period covered and the slow growth rate of PMP, the model required a reductive technique that decreased patient numbers in line with known survival rates. The prevalence calculation method presented here used estimated annual death rates over the 10-year surveillance period to remove non-surviving cases from the cohort each year. Curves reporting overall survival (OS) reflect the total number of patients under surveillance, regardless of disease status. Published OS curves for PMP are relatively uniform, and for a representative estimation of annual death rates, one of the largest PMP cohorts reported was used.^1^ The proportion of patients lost from the cohort each year was manually estimated from the survival curve and the resulting percentages of deceased (cumulative) are presented in Table 1.

**Table 1:** Death rate from pseudomyxoma peritonei, percentage of total patient cohort deceased per year

Year	% Deceased
1	8
2	14
3	20
4	22
5	26
6	29
7	31
8	33
9	35
10	37

### Cure Rate

Cases that had not experienced disease recurrence at year 10 were considered cured and removed from the calculation, and the reported 10-year progression-free survival (PFS) was therefore considered the cure rate. Reported PFS varies according to histological subtype, ranging from 32 to 86% in high- and low-grade subtypes, respectively,^1^,^16^ with low-grade disease being the most commonly diagnosed. In studies where all histological subtypes were included, the low-grade sub-type usually constituted around 70% of cases presenting for surgery.^1^,^7^,^17^ Ten-year PFS of between 47% and 54% was reported for the populations identified within the studies.^1^,^8^,^14^,^18^ For the purpose of the prevalence calculation in this paper, a conservative cure rate of 47% at year 10 was adopted.

## Results

### PMP Incidence Rates in Norway

Based on the statistics obtained from the Norwegian Radium Hospital, the incidence of PMP in Norway was calculated for the years 2009–2018 based on the date of first surgical intervention (Table 2). The number of new people with PMP each year was between 10 and 25, which gave an incidence range of between 2 and 5 per million, with a mean incidence rate of 3.2 people per million per year over the period. The incidence rate of 3.2 people per million was therefore adopted as the base incidence rate used for calculations of prevalence rate.

**Table 2:** PMP incidence rates in Norway and England

Year	Norwegian population data			English population data		
	Population	Number of PMP cases	Incidence rate	Population	Number of PMP cases	Incidence rate
2009	4,779,252	10	2.1			
2010	4,858,199	16	3.3			
2011	4,920,305	10	2.0			
2012	4,985,870	14	2.8	41,726,543	127	3.0
2013	5,051,275	17	3.4	42,059,410	119	2.8
2014	5,109,056	18	3.5	42,460,537	160	3.8
2015	5,165,802	16	3.1	42,861,652	134	3.1
2016	5,213,985	14	2.7	43,265,798	139	3.2
2017	5,258,317	25	4.8	43,559,112	150	3.4
2018	5,337,962	20	3.8			

*PMP* pseudomyxoma peritonei

### PMP Incidence Rates in England

The Peritoneal Malignancy Institute (Basingstoke) treated between 119 and 160 patients from the selected areas per annum between 2012 and 2017, with an annual average of 138 patients. The incidence rate was calculated using the same method as for the Norwegian cohort. The mean incidence rate for England mirrored that in Norway, with 3.2 people per million per annum (Table 2).

### European PMP Incidence

Based on the incidence rate obtained from the Norwegian and British populations, an estimation of the incidence of PMP in Europe was calculated. Table 3 shows the calculated incidence between the years 2009 and 2018 for the European population. In 2018, the estimated incidence for Europe was 1696 people.

**Table 3:** Estimated number of new cases of pseudomyxoma peritonei per year across Europe from 2009 to 2018

Year	Total European population	Incidence
2009	518,299,528	1659
2010	519,963,424	1664
2011	521,489,644	1669
2012	522,872,037	1673
2013	524,147,779	1677
2014	525,373,520	1681
2015	526,585,853	1685
2016	527,803,014	1689
2017	528,999,628	1693
2018	530,116,356	1696

### The Waterfall Prevalence Calculation

Using the incidence rate of 3.2 people per million, a cure rate of 47%, and the death rates shown in Table 1, the prevalence of PMP in Europe was determined using a novel waterfall method designed to capture the overall number of patients living with the disease. The number of patients in the primary cohort each year (“total annual cohort”) was determined using the calculated European incidence (Table 3). The cohort was decayed each year according to the figures noted in Table 1, and at year 10, the 47% were removed from the overall calculation, reflecting the cure rate. Table 4 shows how the waterfall method was applied to determine the prevalence from 2008 to 2018. “Total period cohort” refers to the accumulation of the annual incident cohorts over a 10-year period, i.e. all cases newly diagnosed (Year 1) or continuing to live with the disease each year (Years 2–10) over the decade.

**Table 4:** Aggregative waterfall technique used to create the cascading prevalence chart using data from 2009 to 2018

	2009	2010	2011	2012	2013	2014	2015	2016	2017	2018
Total annual cohort	*1659*	1664	1669	1673	1677	1681	1685	1689	1693	1696
Cured (47%)	**− 780**	− 782	− 784	− 786	− 788	− 790	− 792	− 794	− 796	− 797
Year 1 (− 8%)	*1526*	1531	1535	1539	1543	1547	1550	1554	1557	1561
Year 2 (− 14%)	1421	*1426*	1431	1435	1439	1442	1446	1449	1453	1456
Year 3 (− 20%)	1318	1322	*1327*	1331	1335	1339	1342	1345	1348	1351
Year 4 (− 22%)	1280	1285	1289	*1294*	1298	1302	1305	1308	1311	1314
Year 5 (− 26%)	1209	1214	1219	1223	*1227*	1231	1235	1238	1241	1244
Year 6 (− 29%)	1156	1160	1165	1169	1174	*1178*	1181	1185	1188	1191
Year 7 (− 31%)	1119	1123	1128	1132	1136	1140	*1144*	1148	1151	1155
Year 8 (− 33%)	1082	1086	1091	1095	1099	1103	1107	*1111*	1115	1118
Year 9 (− 35%)	1046	1050	1054	1058	1062	1066	1070	1074	*1078*	1082
Year 10 (− 37%)	1011	1014	1017	1021	1026	1030	1034	1038	1041	*1045*
Total period cohort	12,168	12,212	12,256	12,298	12,339	12,378	12,415	12,450	12,484	12,516
Minus 10-year survivors	11,414	11,455	11,497	11,536	11,574	11,610	11,644	11,676	11,707	**11,736**

Italics follow a single cohort through its iterative cycle; Bold numbers indicate the 47% rate of cure being applied to the full cohort in year 1 and removed from the cohort at the 10th year (total period cohort: accumulation of the annual incident cohorts over a 10-year period)

The actual calculations were initiated in the year 1998; the 10-year time-span between 2009 and 2018 is shown in Table 4. To determine the period prevalence, average total cohorts minus 10-year survivors across the 10-year period were calculated, and the average resulting cohort size was divided by the average European population, giving an overall prevalence rate of 22 people per million, or a total of 11,736 people living with PMP in Europe in 2018.

## Discussion

In this paper, we propose a novel method for period prevalence calculation applied to PMP, which is a slow-growing rare cancer. The method allows a more accurate estimate for diseases where a small difference in incidence has a significant impact on prevalence. We recognise this model is based on a number of assumptions. The incidence rate is of primary importance in prevalence calculations, and can be difficult to determine in rare diseases. Recognition of a disease such as PMP may be delayed by a lack of knowledge and training of clinicians, leading to misdiagnoses and underestimation of incidence, even in countries with specialist centres. In contrast, the risk of overestimation of incidence is negligible.

The incidence rate used for our calculations was based on the number of people undergoing surgery for PMP in Norway and England. Those who were undiagnosed or not referred to the recording site would not be captured, so an incidence of 3.2 per million is probably an underestimate. Using the same waterfall plot calculation method, an incidence adjusted upward by just one per million per year to 4.2 would result in a prevalence of 15,130 people with PMP living in Europe in 2018 whereas the previously assumed maximum incidence of 2 gives a prevalence of 7200 people. This illustrates the importance of establishing accurate incidence figures, including the provision of good quality central registries for rare disease surveillance.

The cure rate is also a key component of the prevalence calculation. A conservative cure rate of 47% was extrapolated from publications where all histological subtypes were included in countries where a curative treatment strategy is well established. The death rates per year were similarly derived from such publications. It is worth noting that the histological subtypes of PMP are associated with very different prognoses, potentially influencing both cure rate and death rate. A consensus for pathology classification of PMP was reached only relatively recently,^19^ and the broader implementation of these diagnostic principles is likely to improve the accuracy of reporting for more countries in the future.

Another influence on the prevalence is the availability of state-of-the-art treatment. In countries where specialist centres do not exist, treatment with curative intent is not likely to be available. This could, in principle, influence prevalence in two ways: either more patients may live with active disease for longer periods of time, leading to *higher* prevalence rates over time, or more patients may lose their lives earlier than would be expected, leading to *lower* prevalence rates. Similarly, the number of prevalent patients could be higher if these patients are offered repeat palliative surgical procedures, leading to longer overall survival, but with no concurrent change to their status as a patient under active care. Ultimately, one could speculate that these influences would balance each other out, but this remains an area of uncertainty.

Incidence rates of “one in a million” seem attractive and imply a rarity that may not reflect reality. Often, understanding prevalence gives a more useful picture of the overall disease burden being experienced by society at large. This allows policy makers to establish effective strategies for diagnosis, treatment and palliation of rare conditions with centralisation of expertise and adequate funding as the NHS does in England through highly specialised commissioning for PMP. The responsibility for collecting accurate incidence data lies with healthcare professionals but needs support from policy makers so that, ultimately, patients benefit.

## Conclusion

In this paper, we have presented a new method for determining the prevalence rates for rare diseases, using the slowly progressive cancer PMP as an example. The adjusted incidence rate of 3.2 people per million per year was based on epidemiological data from Norway and England. A prevalence rate of 22 people per million per year was calculated, which would mean that 11,736 people were living with PMP in Europe in 2018. Although PMP is a rare disease, given its slow and progressive nature, a significant number of people are living with the disease, putting pressure on European health-care systems. Standardising diagnosis and treatment pathways through education and training, sharing knowledge and expertise and establishing European research networks will optimise patients’ outcomes. These prevalence calculations provide a starting point for identifying the burden of disease in this setting and may be a useful tool for similar estimates in other rare diseases. By investing in national and international registries, policy makers can validate these estimates to accurately inform healthcare strategy.
